# Transcriptome Analysis Reveals the Stress Tolerance Mechanisms of Cadmium in *Zoysia japonica*

**DOI:** 10.3390/plants12223833

**Published:** 2023-11-12

**Authors:** Yi Xu, Yonglong Li, Yan Li, Chenyuan Zhai, Kun Zhang

**Affiliations:** 1 College of Grassland Science, Qingdao Agricultural University, Qingdao 266109, China; 20200202793@stu.qau.edu.cn (Y.X.); 20222115004@stu.qau.edu.cn (Y.L.); 20212103050@stu.qau.edu.cn (Y.L.); 20222215010@stu.qau.edu.cn (C.Z.); 2 College of Life Sciences, Qingdao Agricultural University, Qingdao 266109, China

**Keywords:** transcriptome, cadmium, *Zoysia japonica*, differentially expressed genes, molecular mechanism

## Abstract

Cadmium (Cd) is a severe heavy metal pollutant globally. *Zoysia japonica* is an important perennial warm-season turf grass that potentially plays a role in phytoremediation in Cd-polluted soil areas; however, the molecular mechanisms underlying its Cd stress response are unknown. To further investigate the early gene response pattern in *Z. japonica* under Cd stress, plant leaves were harvested 0, 6, 12, and 24 h after Cd stress (400 μM CdCl_2_) treatment and used for a time-course RNA-sequencing analysis. Twelve cDNA libraries were constructed and sequenced, and high-quality data were obtained, whose mapped rates were all higher than 94%, and more than 601 million bp of sequence were generated. A total of 5321, 6526, and 4016 differentially expressed genes were identified 6, 12, and 24 h after Cd stress treatment, respectively. A total of 1660 genes were differentially expressed at the three time points, and their gene expression profiles over time were elucidated. Based on the analysis of these genes, the important mechanisms for the Cd stress response in *Z. japonica* were identified. Specific genes participating in glutathione metabolism, plant hormone signal and transduction, members of protein processing in the endoplasmic reticulum, transporter proteins, transcription factors, and carbohydrate metabolism pathways were further analyzed in detail. These genes may contribute to the improvement of Cd tolerance in *Z. japonica*. In addition, some candidate genes were highlighted for future studies on Cd stress resistance in *Z. japonica* and other plants. Our results illustrate the early gene expression response of *Z. japonica* leaves to Cd and provide some new understanding of the molecular mechanisms of Cd stress in *Zosia* and *Gramineae* species.

## 1. Introduction

With industrial development, Cadmium (Cd) has become a major heavy metal pollutant and extremely toxic to plants and humans [1]. Cd ions are often taken up into the airflow layer and released into the soil through atmospheric sedimentation, rainfall, and snowfall [2]. In addition, agricultural sewage irrigation, which contains a Cd solution, also increases the soil Cd ion content [3]. Previous strategies to remediate heavy metal pollution have mainly focused on physical and chemical methods [4]. However, these methods are neither durable nor reliable. Nowadays, phytoremediation is an eco-friendly approach that could remediate soil Cd contamination. It has multiple benefits, including a low cost, safety, reliability, and minimal environmental interference [5,6]. Actually, because of the food security problem for humans, not all plants are suitable for phytoremediation under Cd contamination aera. However, turfgrass could be a good candidate plant for phytoremediation in heavy metal-contaminated regions with great degradation ability and ornamental value [7,8]. 

Cd is an ion element that is nonessential for the growth and development of plants. Many studies have shown that Cd absorption can induce serious cellular damage, and that multiple physiological and metabolic processes interfere with plant growth [9]. Plants have evolved many detoxification mechanisms to reduce or resist Cd toxicity, including Cd ion absorption, chelation with metabolites, vacuolar storage, the activation of antioxidant systems, and related gene expression regulation networks [10]. When Cd enters the cell, cell plasma membrane proteins, such as ZRT, the IRT-like protein family protein, the natural resistance-associated macrophage protein, and yellow stripe-like (YSL) proteins, limit intracellular and extracellular Cd transportation [11]. The ATP-binding cassette transporter and heavy metal ATPase families of genes can be expressed in vacuoles, and Cd ions can be compartmentalized in roots and leaves to reduce Cd ion hyperaccumulation and protect plants [12]. Metallothionein, phytochelatin, glutathione, and pectin are the major heavy metal chelators in plants [13]. When the Cd ion concentration in the cytoplasm increases, these metabolites fix the Cd ions to chelate them in vacuoles or cell walls. In addition, antioxidant systems, including superoxide dismutase, catalase, ascorbate peroxidase, glutathione peroxidase, dehydroascorbate reductase, and glutathione reductase, play important roles in scavenging reactive oxygen species during Cd stress [14]. Meanwhile, the above-mentioned physiological and metabolic processes, which are regulated by a complex network system involving a large number of genes, are the core components of the plant Cd stress detoxification [15]. 

Transcription factors play an important regulatory role in plant development and their response to various environmental stresses. Under Cd stress conditions, transcription factors participate in the plant Cd stress response and tolerance by controlling the expression of downstream genes [16]. Numerous transcription factors, such as bHLH, MYB, and WRKY, have been reported to be involved in plant Cd stress tolerance. For example, *AtMYB4* overexpression can improve plant Cd resistance by specifically activating phytochelatin synthases and metallothionein 1C gene expression and promoting phytochelatin and metallothionein synthesis for plant detoxification [17]. Cd stress can upregulate the expression of the OBP3-responsive gene (ORG), which is a bHLH transcription factor in soybean (*Glycine max*), and *GmORG3* overexpression enhances cadmium tolerance via increasing iron and reducing cadmium uptake from roots to shoots [18]. Cd stress could also induce the expression of *WRKY45*. Overexpressing *WRKY45* confers Cd tolerance via promoting the expression of phytochelatin-synthesis-related genes, *PCS1* and *PCS2*, respectively, in Arabidopsis [19]. In addition, other transcription factors have been reported to play important roles in the signal transduction of a Cd stress response by regulating a series of genes involved in Cd uptake, transport, and tolerance in different plant such as tea plants (*Camellia sinensis*) [20], muskmelon [21], and the golden rain tree (*Koelreuteria paniculata*) [22].

*Zoysia japonica* is an important perennial warm-season turf grass with a large biomass and excellent abiotic stress resistance [23]. A previous study has shown the potential ability of *Z. japonica* as a bioindicator in heavy metal pollution areas [24], but its molecular mechanisms regarding Cd tolerance are not yet well understood, which provides a limitation for *Z. japonica* utilization in soil remediation in Cd-contaminated areas. In the present study, we characterized the differentially expressed genes in *Z. japonica* leaves under Cd stress at different time points and explored the tolerance mechanisms of *Z. japonica* in response to Cd toxicity. Our results are an important addition to plant Cd stress molecular mechanism studies and may contribute to improvements in the phytoremediation and environmental protection of soils polluted with heavy metals. Moreover, the novel candidate genes identified in our study may serve as molecular markers for further improvement of Cd stress tolerance in *Zoysia* and other grass species.

## 2. Results

### 2.1. Transcriptome Analysis in Z. japonica Leaves after Cd Treatment

In the present study, *Z. japonica* leaves were harvested 0, 6, 12, and 24 h after Cd stress treatment and subjected to RNA sequencing (RNA-seq) analysis. Twelve cDNA libraries were constructed from *Z. japonica* leaves and sequenced following Cd stress treatment. The RNA-seq data were uploaded to the NCBI SRA database (no. PRJNA1008854). For each library, 39,850,416–46,067,222 and 39,253,016–45,438,370 bp of raw and clean reads were generated, respectively (Table 1). The total mapped rate was higher than 94%, with more than 6,017,412,816 bp of sequence. The Q20 and Q30 values exceeded 97% and 93%, respectively. In addition, a strong correlation was observed between the different samples (Figure S1). These results indicated that the RNA-seq data were of high quality.

### 2.2. Differential Gene Expression Analysis in Z. japonica Leaves under Cd Treatment

**Figure 1:**
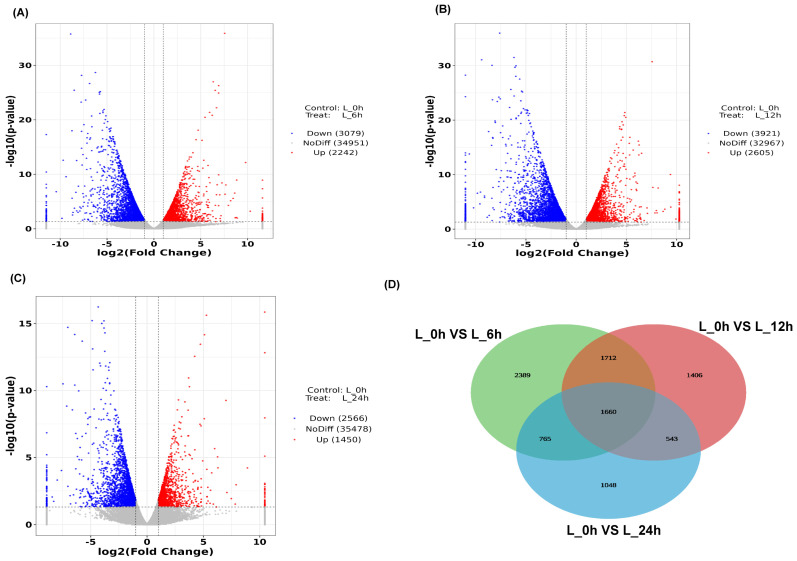
The profile of differentially expressed genes at different time points. (**A**) Volcano plot of the L_6 h vs. L_0 h group; (**B**) Volcano plot of the L_12 h vs. L_0 h group; (**C**) Volcano plot of the L_24 h vs. L_0 h group; (**D**) Venn diagram for all differentially expressed genes.

Differentially expressed genes were screened using the standard cut-off metrics of |log2 fold change| > 1 and a *p*-value < 0.05. Volcano plots identified 5321, 6526, and 4016 genes expressed 6, 12, and 24 h after Cd stress treatment, respectively (Figure 1). This indicates that a strong induction of gene expression occurred 12 h after Cd stress treatment. Between L_6 h (leaves sampled 6 h after Cd treatment) and L_0 h (control), 2242 and 3097 upregulated and downregulated genes, respectively, were identified (Figure 1A); between L_12 h (leaves sampled 12 h after Cd treatment) and L_0 h, 2605 and 3921 upregulated and downregulated genes, respectively, were identified (Figure 1B); and between L_24 h (leaves sampled 24 h after Cd treatment) and L_0 h, 1450 and 2566 upregulated and downregulated genes, respectively, were identified (Figure 1C). 

For L_6 h vs. L_0 h group, a gene ontology (GO) analysis showed that the chloroplast, plastid, thylakoid, plastid envelope, plastid stroma, plastid thylakoid, chloroplast thylakoid, chloroplast stroma, plastid membrane, and chloroplast thylakoid membrane were the top ten GO terms in the cellular component category (*p* < 0.05). Photosynthesis, photosynthesis light reaction, photosynthetic electron transport chain, plastid organization, generation of precursor metabolites and energy, chloroplast organization, response to abiotic stimulus, response to light intensity, electron transport chain, and photosynthetic electron transport in photosystem I were the top ten GO terms in biological processes. For the molecular function category, phosphoenolpyruvate carboxykinase activity, protein-domain-specific binding, snoRNA binding, sugar-phosphatase activity, inorganic molecular entity transmembrane transporter activity, starch synthase activity, carbon–carbon lyase activity, inorganic diphosphate phosphatase activity, fructose 1,6-bisphosphate 1-phosphatase activity, and transmembrane transporter activity were highly enriched in the *Z. japonica* leaves under Cd stress treatment (Figure S2A).

For the L_12 h vs. L_0 h group GO analysis, the plastid stroma, chloroplast stroma, chloroplast, plastid, plastid thylakoid membrane, chloroplast thylakoid membrane, thylakoid, photosynthetic membrane, thylakoid membrane, and plastid envelope were the top ten GO terms in the cellular component category (*p* < 0.05). Photosynthesis, the response to abiotic stimulus, photosynthesis light reaction, glucose metabolic process, gluconeogenesis, hexose biosynthetic process, response to karrikin, response to light stimulus, hexose metabolic process, and rRNA metabolic process were the top ten GO terms in the biological process category. For the molecular function category, snoRNA binding, phosphoenolpyruvate carboxykinase activity, fructose 1,6-bisphosphate 1-phosphatase activity, oxidoreductase activity, carbon–carbon lyase activity, pigment binding, sugar-phosphatase activity, transmembrane transporter activity, catalytic activity, inorganic molecular entity, and transmembrane transporter activity were highly enriched in *Z. japonica* leaves under Cd stress treatment (Figure S2B).

For the L_24 h vs. L_0 h group GO analysis, the chloroplast, plastid, plastid envelope, plastid stroma, chloroplast stroma, thylakoid, organelle envelope, envelope, chloroplast envelope, and plastid membrane were the top ten GO terms in the cellular component category (*p* < 0.05). Photosynthesis, the photosynthesis light reaction, plastid organization, chloroplast organization, generation of precursor metabolites and energy, glucose metabolic process, starch metabolic process, photosynthetic electron transport chain, cellular glucan metabolic process, and hexose biosynthetic process were the top ten GO terms in the biological process category. For the molecular function category, phosphoenolpyruvate carboxykinase activity, fructose 1,6-bisphosphate 1-phosphatase activity, sugar-phosphatase activity, transmembrane transporter activity, inorganic molecular entity transmembrane, transporter activity, carbon–carbon lyase activity, oxidoreductase activity, oxidoreductase activity, acting on other nitrogenous compounds as donors, and ion transmembrane transporter activity were highly enriched in the *Z. japonica* leaves under Cd stress treatment (Figure S2C).

For the KEGG pathway analysis, the pathways associated with carbon fixation in photosynthetic organisms; photosynthesis; photosynthesis-antenna proteins; nitrogen metabolism; pyruvate metabolism; glyoxylate and dicarboxylate metabolism; pentose phosphate pathway; glycolysis/gluconeogenesis; glycine, serine, and threonine metabolism; and alanine, aspartate, and glutamate metabolism were the ten most enriched in the L_6 h vs. L_0h group (Figure S3A). For the L_12 h vs. L_0h group, carbon fixation in photosynthetic organisms, photosynthesis-antenna proteins, glycolysis/gluconeogenesis, pyruvate metabolism, ribosome biogenesis in eukaryotes, photosynthesis, pentose phosphate pathway, nitrogen metabolism, flavonoid biosynthesis, and glyoxylate and dicarboxylate metabolism were the ten most enriched KEGG pathways (Figure S3B). For the L_24 h vs. L_0h group, carbon fixation in photosynthetic organisms, starch and sucrose metabolism, pyruvate metabolism, nitrogen metabolism, glycolysis/gluconeogenesis, pentose phosphate pathway, phenylalanine metabolism, photosynthesis, flavonoid biosynthesis, and ubiquinone and other terpenoid-quinone biosyntheses were the ten most enriched KEGG pathways (Figure S3C). 

### 2.3. Common Differentially Expressed Genes Identified in the Time Course Analysis

**Figure 2:**
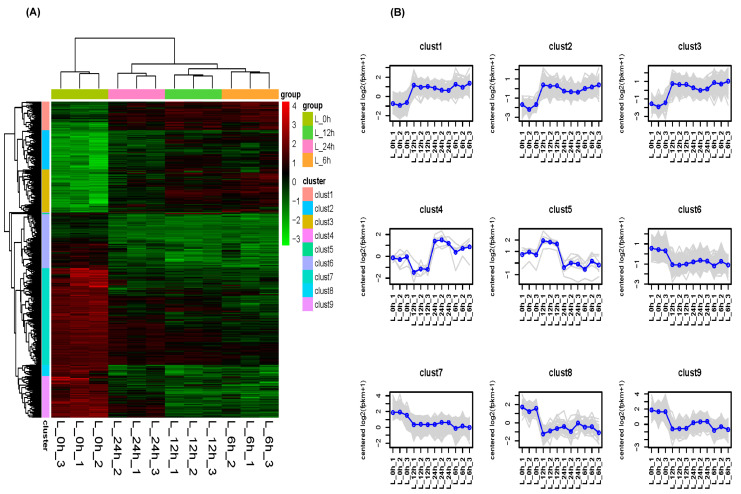
Expression profiles of the common genes at different time points. (**A**) Heatmap of all differentially expressed genes; (**B**) cluster analysis of differentially expressed gene profiles.

The Venn diagram in Figure 1D shows that there were 1660 genes differentially expressed in the L_6 h vs. L_0 h, L_12 h vs. L_0 h, and L_24 h vs. L_0 h groups. These common differentially expressed genes play an important role in the response of *Z. japonica* to Cd stress and were chosen for further analysis (Table S2). The variation in the common differentially expressed genes is displayed in Figure 2A. The dynamic expression patterns of the differentially expressed genes were analyzed. The 1660 differentially expressed genes were divided into nine clusters (Figure 2B), including three upregulated clusters (clusters 1, 2, and 3) and four downregulated clusters (clusters 6, 7, 8, 9). 

**Figure 3:**
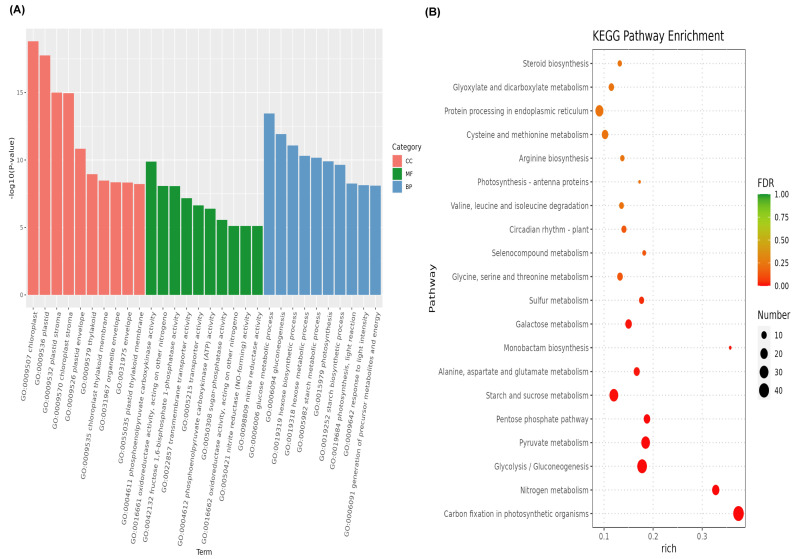
The GO and KEGG enrichment analysis of commonly expressed genes. (**A**) GO analysis; (**B**) KEGG enrichment analysis.

The common differentially expressed genes were subjected to a GO analysis (Figure 3A, Table S3). These genes were mainly involved in the chloroplasts, plastids, plastid stroma, chloroplast stroma, plastid envelope, thylakoid, chloroplast thylakoid membrane, organelle envelope, envelope, and plastid thylakoid membrane in the cellular component category (*p* < 0.05). The glucose metabolic process, gluconeogenesis, the hexose biosynthetic process, the hexose metabolic process, the starch metabolic process, photosynthesis, the starch biosynthetic process, the photosynthesis light reaction, the response to light intensity, and the generation of precursor metabolites and energy were the top ten GO terms in the biological process category. In the molecular function category, phosphoenolpyruvate carboxykinase activity, oxidoreductase activity, acting on other nitrogenous compounds as donors, fructose 1,6-bisphosphate 1-phosphatase activity, transmembrane transporter activity, transporter activity, phosphoenolpyruvate carboxykinase (ATP) activity, sugar-phosphatase activity, oxidoreductase activity, cytochrome as an acceptor, and nitrite reductase (NO-forming) activity were highly enriched in the *Z. japonica* leaves under Cd stress treatment. The common differentially expressed genes were also subjected to a KEGG pathway analysis. As shown in Figure 3B and Table S4, the pathways associated with carbon fixation in photosynthetic organisms; nitrogen metabolism; glycolysis/gluconeogenesis; pyruvate metabolism; the pentose phosphate pathway; starch and sucrose metabolism; alanine, aspartate, and glutamate metabolism; monobactam biosynthesis; and galactose metabolism were the top ten significantly enriched pathways in the *Z. japonica* leaves under Cd stress treatment.

### 2.4. The Molecular Mechanism of the Response to Cd Treatment in Z. japonica Leaves

**Figure 4:**
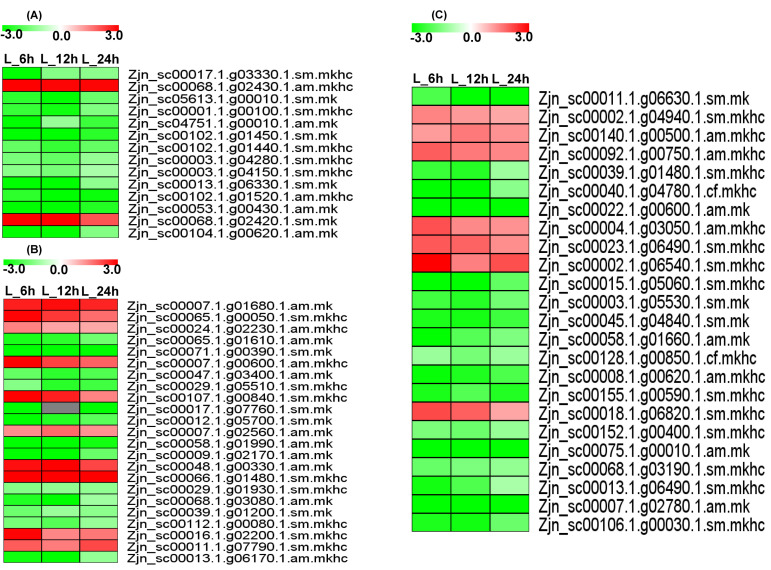
Expression profiles of commonly expressed genes related to glutathione metabolism, plant hormone signal and transduction, and transcription regulation pathways. (**A**) Glutathione metabolism pathway; (**B**) plant hormone signal and transduction pathway; (**C**) Protein processing in endoplasmic reticulum pathway.

Briefly, the molecular mechanism was composited by the following processes. Fourteen glutathione-metabolism-related genes were commonly differentially expressed in the L_6 h vs. L_0 h, L_12 h vs. L_0 h, and L_24 h vs. L_0 h groups (Figure 4A, Table S5). Most of the differentially expressed genes were downregulated. However, one glutathione transferase, *GST23* (Zjn_sc00068.1.g02430.1.am.mkhc) (3.95-, 2.94, and 2.95-fold), and one probable glutathione S-transferase (Zjn_sc00068.1.g02420.1.sm.mk) (4.23-, 3.57-, and 2.03-fold) were upregulated in the L_6 h vs. L_0 h, L_12 h vs. L_0 h, and L_24 h vs. L_0 h groups, respectively, indicating that these two genes have potential roles in Cd chelation mediated by glutathione.

A total of 23 plant hormone-signal-transduction-related genes (10 upregulated and 13 downregulated) were commonly differentially expressed between the L_6 h vs. L_0h, L_12 h vs. L_0 h, and L_24 h vs. L_0 h groups (Figure 4B, Table S6). Among these genes, auxin-responsive proteins (Zjn_sc00007.1.g01680.1.am.mk, Zjn_sc00066.1.g01480.1.sm.mkhc, Zjn_sc00024.1.g02230.1.am.mkhc, Zjn_sc00107.1.g00840.1.sm.mkhc) (*IAA9*, *IAA15*, and *IAA21*), ethylene receptor 3 (Zjn_sc00007.1.g00600.1.am.mkhc), the abscisic acid receptor (Zjn_sc00016.1.g02200.1.sm.mkhc) (*PYL5*), and the probable protein phosphatase 2C 9 (Zjn_sc00048.1.g00330.1.am.mk) were upregulated, suggesting that these hormone signal transduction pathways play roles in Cd detoxification.

Twenty-four differentially expressed genes (7 upregulated and 17 downregulated) were involved in protein processing in the endoplasmic reticulum pathway (Figure 4C, Table S7). These included the probable ubiquitin receptor (Zjn_sc00018.1.g06820.1.sm.mkhc) (*RAD23*); the dolichyl-diphosphooligosaccharide-protein glycosyltransferase subunit (Zjn_sc00023.1.g06490.1.sm.mkhc) (*STT3B*); the eIF-2-alpha kinase (Zjn_sc00004.1.g03050.1.am.mkhc) (*GCN2*); protein disulfide isomerase (Zjn_sc00140.1.g00500.1.am.mkhc), and the eukaryotic translation initiation factor 2 subunit alpha homolog (Zjn_sc00002.1.g04940.1.sm.mkhc), which were upregulated, suggesting that different protein or enzyme metabolism pathways are involved in Cd stress tolerance. 

**Figure 5:**
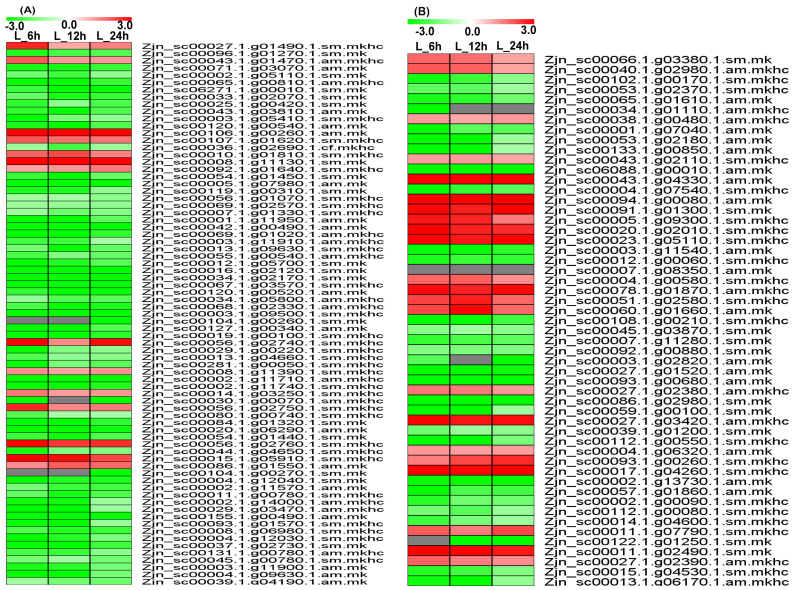
Expression profiles of common expressed genes related to transportation and protein processing in endoplasmic reticulum pathway. (**A**) Transporter protein genes; (**B**) transcription factor.

A total of 75 differentially expressed genes (14 upregulated and 61 downregulated) were identified with transport functions (Figure 5A, Table S8), including a sugar transporter ERD6-like 4/5 (Zjn_sc00027.1.g01490.1.sm.mkhc, Zjn_sc00043.1.g01470.1.am.mkhc); probable metal-nicotianamine transporters (Zjn_sc00056.1.g02740.1.sm.mkhc, Zjn_sc00056.1.g02750.1.sm.mkhc, Zjn_sc00056.1.g02760.1.sm.mkhc) (*YSL12*, *YSL13*); a probable mitochondrial adenine nucleotide transporter (Zjn_sc00015.1.g05910.1.sm.mkhc) (*BTL1*); sugar transport protein 14 (Zjn_sc00107.1.g01620.1.sm.mkhc); oligopeptide transporter 1 (Zjn_sc00106.1.g00260.1.am.mk); equilibrative nucleotide transporter 3 (Zjn_sc00008.1.g11390.1.sm.mkhc); the adenine nucleotide transporter (Zjn_sc00086.1.g01550.1.am.mk) (*BT1*); and folate transporter 1 (Zjn_sc00014.1.g03250.1.sm.mkhc), indicating that the transportation of Cd ions and small-molecule metabolic substances was aggregated in *Z. japonica.*

Fifty-three transcription-factor-related genes (23 upregulated and 30 downregulated) were commonly differentially expressed between the L_6 h vs. L_0 h, L_12 h vs. L_0 h, and L_24 h vs. L_0 h groups (Figure 5B, Table S9). These genes included basic helix–loop–helix transcription factors (Zjn_sc00038.1.g00480.1.am.mkhc, Zjn_sc00007.1.g08350.1.am.mk) (*bHLH74*, *bHLH96*), ethylene-responsive transcription factors (Zjn_sc00004.1.g06320.1.am.mk, Zjn_sc00043.1.g04330.1.am.mk, Zjn_sc00027.1.g03420.1.am.mkhc, Zjn_sc00078.1.g01870.1.am.mkhc) (*ERF1A*, *CRF1*, WRI1, *ABR1*), heat stress transcription factors (Zjn_sc00020.1.g02010.1.sm.mkhc, Zjn_sc00017.1.g04260.1.sm.mkhc, Zjn_sc00011.1.g02490.1.sm.mk) (*HSFA3*, *HSFC1B*), transcription factor *ICE1* (Zjn_sc00094.1.g00080.1.am.mk) (*SCRM*), a MADS-box transcription factor (Zjn_sc00091.1.g01300.1.sm.mk) (*MADS23*), and transcription factor *GAMYB* (Zjn_sc00040.1.g02980.1.am.mkhc) (*GAM1*), which were upregulated and suggested to play important roles in transcriptional regulation in response to Cd stress.

In addition, 91 carbohydrate-metabolism-related genes (six upregulated and eighty-five downregulated) were identified (Table S10), but only six of these genes, including benzaldehyde dehydrogenase (Zjn_sc00007.1.g05950.1.sm.mkhc), plastidial pyruvate kinase 2 (Zjn_sc00036.1.g00110.1.am.mkhc), dihydrolysine residue acetyltransferase component 4 of the pyruvate dehydrogenase complex (Zjn_sc00091.1.g01210.1.sm.mkhc), and phosphoglycerate mutase-like protein 4 (Zjn_sc00028.1.g03020.1.sm.mkhc), were upregulated after the Cd stress treatment, indicating that the carbohydrate-related pathway was severely inhibited by Cd toxicity.

**Figure 6:**
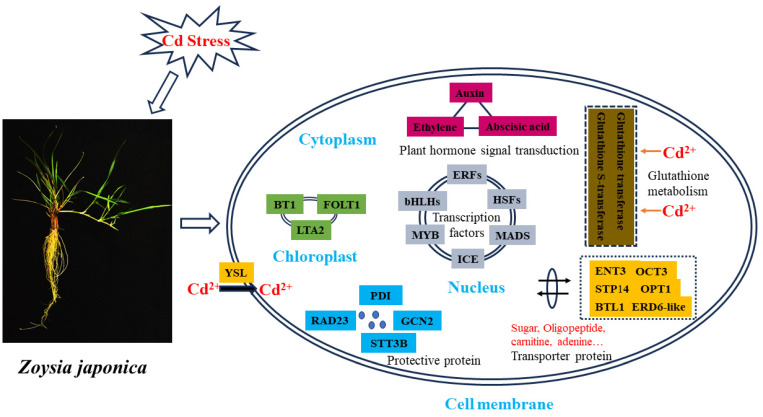
A simple diagram associated with the Cd tolerance mechanism in *Z. japonica* leaves. Basic helix–loop–helix transcription factors, bHLH; adenine nucleotide transporter, BT1; probable mitochondrial adenine nucleotide transporter, BTL1; Equilibrative nucleotide transporter 3, ENT3; ethylene-responsive transcription factors, ERFs; Sugar transporter ERD6-like, ERD6-like; Folate transporter 1, FOLT1; eIF-2-alpha kinase, GCN2; heat stress transcription factors, HSFs; ICE-like transcription factor, ICE; dihydrolipoyllysine-residue acetyltransferase component 4 of the pyruvate dehydrogenase complex, LTA2; MADS-box transcription factor, MADS; organic cation/carnitine transporter 3, OCT3; oligopeptide transporter 1, OPT1; protein disulfide isomerase, PDI; probable ubiquitin receptor, RAD23; sugar transport protein 14, STP14; dolichyl-diphosphooligosaccharide-protein glycosyltransferase subunit, STT3B; probable metal-nicotianamine transporters, YSLs.

Figure 6 shows a simple graph of the Cd tolerance mechanism in *Z. japonica* leaves. When Cd ions are transported into the cell cytoplasm, transcriptional regulation begins, and different metabolic processes, such as protein metabolism, glutathione metabolism, and carbohydrate metabolism, are triggered. Hormones, such as auxin, ethylene, and abscisic acid, participate in Cd detoxification. 

### 2.5. Validation of RNA-seq Results with qRT-PCR

**Figure 7:**
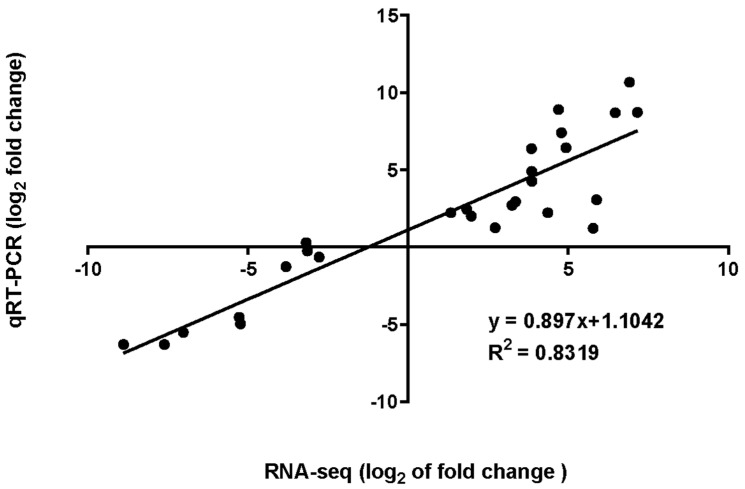
Validation of RNA-seq results with qRT-PCR.

To verify the RNA-seq results, we randomly selected nine differentially expressed genes and performed a quantitative reverse transcription polymerase chain reaction (qRT-PCR) analysis of the L_6 h vs. L_0 h, L_12 h vs. L_0 h, and L_24 h vs. L_0 h groups. The relative expression levels of the differentially expressed genes are shown in Table S11. We then performed a correlation analysis of the RNA-seq and qRT-PCR results (Figure 7). A strong correlation (R^2^ = 0.8319) was observed, confirming the reliability of the RNA-seq data. 

## 3. Discussion

RNA-seq has been widely used to explore the molecular mechanisms underlying Cd stress in plants, especially in Cd-accumulating species. For example, 110.07 Gb of clean data and 63,957 unigenes were acquired from the hyperaccumulator *Phytolacca americana* after the Cd stress treatment [25]. In the Cd accumulator *Erigeron canadensis*, 12 cDNA libraries were constructed, and a total of 89.51 Gb of clean reads were obtained after 0.5 mmol/L of Cd treatment [26]. In the hyperaccumulator plant *Noccaea caerulescens*, transcriptomes were assembled using the Trinity de novo assembler and annotated along with the predicted protein sequences [27]. Turf grass is an important phytoremediation plant as it successfully mitigates soil Cd levels during the cutting and regrowth processes [28]. However, few studies have performed an RNA-seq analysis of Cd stress tolerance in turf grass species, and only in seashore paspalum (*Paspalum vaginatum*) and tall fescue (*Festuca arundinacea*) [29,30]. In this study, we obtained high-quality transcriptome data that can provide new insights into the plant Cd stress tolerance mechanisms in turfgrass species. 

Glutathione plays an important role in the phytochelatin pathways in plants that cope with cadmium stress [31]. Under Cd stress, the expression of glutathione-metabolism-related genes is induced, and this promotes the biosynthesis of phytochelatin to chelate free Cd to generate a Cd–phytochelatin complex, which is then transported to the vacuole to isolate and complete the detoxification of Cd [32]. Increased glutathione metabolism has been reported in *Solanum nigrum* and clove basil *(Ocimum gratissimum)* under Cd stress conditions [33,34]. One glutathione transferase and one probable glutathione S-transferase were found to be upregulated in the present study, suggesting that these genes are closely associated with Cd detoxification in *Z. japonica* leaves. 

Under Cd stress, plant hormones (such as ABA and IAA) undergo significant changes, and the expression levels of antioxidant synthesis and transport protein genes are affected [35]. ABA treatment counteracts Cd-induced fluctuations in non-enzymatic proteins and antioxidant enzymes [36]. The abscisic acid receptor PYL5 and probable protein phosphatase 2C 9 were upregulated after Cd stress, suggesting that the ABA signaling pathway was activated. Previous studies have shown that there are significant differences in the IAA concentration and distribution in the primary root tips and cotyledons of Cd-treated plants [37]. IAA content is significantly reduced after Cd treatment. In rice (*Oryza sativa*) shoots, GO and KEGG enrichment analyses showed that genes encoding the auxin-responsive protein IAA were upregulated after Cd stress [38]. Genes encoding the auxin-responsive proteins IAA9, 15, and 21 were upregulated in the present study, suggesting that auxins are involved in the response of *Z. japonica* leaves to Cd stress.

The endoplasmic reticulum is a complex metabolic site and an important organelle for the regulation of protein synthesis, signal transduction, and calcium homeostasis [39]. The synthesis and folding of all secreted proteins and most membrane proteins, as well as the modification and processing of proteins (mainly glycosylation, hydroxylation, acylation, and disulfide bond formation), are carried out in the endoplasmic reticulum. When plants are stressed by heavy metals, large amounts of unfolded or misfolded proteins accumulate in cells, resulting in endoplasmic reticulum stress [40]. The probable ubiquitin receptor RAD23 has been reported to be a principal shuttle of ubiquitin conjugates and to contribute to Arabidopsis UV tolerance [41]; the dolichyl-diphosphooligosaccharide-protein glycosyltransferase subunit STT3B could encode an essential subunit of the oligosaccharyltransferase complex and control adaptive responses to stress in plants [42]; the eIF-2-alpha kinase GCN2 functions as a translation of mRNAs and plays a role in plants’ stress responses [43]; protein disulfide isomerase had been reported to participate in plant abiotic and biotic stress resistance [44]. These genes, which relate to protein processing in the endoplasmic reticulum pathway, were upregulated after the Cd treatment in the present study. However, the potential role of these genes in regulating Cd stress tolerance requires further analyses.

The oligopeptide transporter (OPT) family plays diverse roles in metal homeostasis, nitrogen mobilization, and sulfur distribution [45]. In *Arabidopsis*, 17 OPT members have been identified and phylogenetically divided into two subfamilies: OPT and YSL [46]. Many OPTs have been shown to be involved in the uptake and translocation of Fe and Cd [47]. OPT1 was also identified in the present study, suggesting that it plays an important role in metal homeostasis. The YSL family plays an important role in the transport of metal ions and in chelating metals (Fe, Cu, Mn, and Cd) [48]. Feng et al. discovered that SnYSL3 encodes a local plasma transporter that transports multiple metal–nicotinamide complexes and that SnYSL3 plays an important role in Cd stress [49]. In blueberries, VcYSL6 is upregulated by Cd stress, and its overexpression increases the Cd accumulation in the leaves [50]. Two probable metal–nicotianamine transporters were found to be upregulated in our study, suggesting an important role for nicotianamine chelation via *YSL* genes in *Z. japonica*. Sugar transporters mediate sugar transport in plants and play crucial roles in various physiological processes [51]. Here, we found that the sugar transporters ERD6-like 4/5 and sugar transport protein 14 showed increased expression levels after Cd stress, which is consistent with the KEGG analysis that showed that sugar-related pathways were the most enriched pathways under Cd stress conditions. 

Transcription factors bind to DNA and are involved in the activity of hormonal and antioxidant enzymes, thereby regulating the absorption, transport, and accumulation of Cd. *ERF* gene expression plays a crucial role in the Cd stress response in plants. For example, in *Hibiscus syriacus*, numerous ERF-family transcription factors were induced after Cd stress [52]. In creeping bentgrass (*Agrostis stolonifera*), ERF1B, ERF110, ERF7, ERF113, and ERF15 are upregulated in response to Cd stress [53]. In *Iris lacteal*, the overexpression of *IlAP2*, an AP2/ERF superfamily gene, reduced Cd toxicity by hindering Cd transport [54]. In the present study, ethylene-responsive transcription factors (*ERF1A*, *CRF1*, *WRI1*, and *ABR1*) were upregulated after Cd treatment, suggesting that they may play critical roles in the Cd stress tolerance of *Z. japonica*. Heat shock transcription factors (HSFs) regulate HSP expression, participate in various aspects of protein homeostasis (such as the refolding, assembly, and transport of damaged proteins), and maintain protein stability [55]. Previous studies have proven that Cd stress could lead to the upregulation of *HSF* family genes and metallothionein genes in wheat and rice roots [56]. HSF3A and HSFC1B have been widely identified as playing roles in plant oxidative stress tolerance, and HSFC1B is induced by salt and heat stress [57]. HSF3A and HSFC1B from the *Z. japonica* leaves were found to be upregulated after the Cd stress treatment in this study, implying their potentially novel functions in Cd tolerance in *Z. japonica*. bHLH transcription factors are involved in the regulation of plant growth and development [58]. bHLH74 is required for leaf and root development in *Arabidopsis* [59]. In *Neolamarckia cadamba*, bHLH74 has been reported to function in cell elongation and is upregulated after gibberellin treatment [60]. In tomatoes, SlbHLH96 interacts with SlERF4 and represses the expression of ABA catabolic genes, thereby promoting drought tolerance [61]. In rice, OsbHLH96 influences brown planthopper resistance by regulating the expression of pathogen-related genes [62]. In the present study, bHLH74 and bHLH96 were found to be upregulated after Cd stress treatment, implying that these two transcription factors may positively regulate the Cd stress response in *Z. japonica* leaves by affecting plant growth, development, and stress-related gene expression.

Previous studies have shown that Cd stress affects the chlorophyll content and carbon metabolism enzyme activity in a plant’s photosynthetic organs and destroys the photosynthetic structure of plants, thereby reducing their photosynthetic rate. Cd reduces the photosynthetic efficiency of plants by affecting photoreactions, dark reactions, and photochemical efficiencies [63,64]. Similar results were observed in this study, which found that carbon fixation in photosynthetic organisms was the most enriched pathway in the KEGG analysis, and most carbohydrate-metabolism-related genes were downregulated after the Cd stress treatment, suggesting that the photosynthetic process was inhibited. 

In summary, this study revealed the molecular mechanisms of the response of *Z. japonica* leaves to Cd stress at different time points. A series of specific genes in glutathione metabolism, plant hormone signaling and transduction, and those involved in protein processing in the endoplasmic reticulum, transporter proteins, transcription factors, and carbohydrate metabolism pathways were highlighted in *Z. japonica*. Candidate genes, including the *bHLH*, *ERF*, and *HSF* family genes; *OPT* genes; *GST* genes; and auxin-responsive protein genes, which are shown in Figure 6, are novel for a plant Cd stress tolerance study. A similar abiotic stress regulation function of these novel genes has been reported in Arabidopsis or other model plants, which suggests their potential and special roles during Cd detoxification in *Z. japonica*. Therefore, further experiments on how the function of these genes varies in plant Cd stress tolerance should be carried out. Overall, our results enrich the understanding of the molecular mechanisms of Cd stress in *Z. japonica* and also provide important clues for Cd tolerance genetic breeding in other turfgrass and Gramineae species. 

## 4. Materials and Methods

### 4.1. Plant Materials and Methods

*Z. japonica* seeds (Shi Ji Tian Yuan Ecological Technology Co., Zhengzhou, China) were sown in fritted clay and watered with a half-strength Hoagland nutrient solution. After approximately 3 months of cultivation in a growth chamber (GXZ-500, Jiangnan, China) (25/20 °C (day/night temperature), 16 h light (1200 μmol m^−2^s^−1^)/8 h dark), uniform *Z. japonica* seedlings were selected and transferred to hydroponic conditions with half-strength Hoagland. Plants were cultivated for 2 months and then subjected to Cd treatment. The Cd concentration was 400 μM, supplied as CdCl_2_ based on the previous study [65]. Leaf tissues were sampled 0, 6, 12, and 24 h after Cd treatment for physiological measurements, RNA isolation, and RNA-seq analysis. All time points had three biological replicates.

### 4.2. Total RNA Isolation and Sequencing Library Generation

Total RNA was isolated using a MiniBEST Plant RNA Extraction Kit (Takara, Kusatsu, Japan). RNA concentration, quality, and integrity were determined using a NanoDrop spectrophotometer (Thermo Fisher Scientific, Waltham, MA, USA). Three micrograms of RNA were used for sequencing library generation using a previously published protocol. The library was sequenced using a NovaSeq 6000 platform (Illumina, San Diego, CA, USA).

### 4.3. Quality Control and Read Mapping

The original (raw) sequencing data were exported in an FASTQ format. The raw data contained a number of adaptor sequences and low-quality reads; therefore, Cutadapt (v1.15) software was used to filter the sequencing data to obtain high-quality (clean) reads for further analysis. The reference genome and gene annotation files were downloaded from the *Zoysia* Genome Database (http://zoysia.kazusa.or.jp/ (accessed on 1 June 2023)). The filtered reads were mapped to the reference genome using HISAT2 v2.0.5. 

### 4.4. Differential Expression Analysis

HTSeq (0.9.1) statistics were used to compare the read count values for each gene with the original gene expression data and then to standardize the expression levels. Differences in gene expression levels were analyzed using DESeq (version 1.30.0) with screening conditions as follows: expression difference multiple |log2 fold change| > 1 and *p*-value < 0.05. An R language software package, Pheatmap (1.0.8), was used to perform a bidirectional clustering analysis of all the differentially expressed genes in the samples. Heatmaps were generated according to the expression levels of the same gene in different samples and the expression patterns of different genes in the same sample, using the Euclidean method to calculate the distance and the complete linkage method for clustering. The dynamic expression patterns of the differentially expressed genes were analyzed using Short Time-series Expression Miner software (v1.3.8) [66].

### 4.5. GO and KEGG Enrichment Analyses

The mapped genes were further analyzed using the GO database, and the number of differentially expressed genes in each term was calculated. topGO was used to perform a GO enrichment analysis of the differentially expressed genes, and the *p*-value was calculated using the hypergeometric distribution method (the standard of significant enrichment is a *p*-value < 0.05). The GO terms significantly enriched for the differentially expressed genes were used to determine the main biological functions performed by the genes. ClusterProfiler (v3.4.4) software was used to perform a KEGG pathway enrichment analysis of the differentially expressed genes, focusing on significantly enriched pathways with a *p*-value < 0.05.

### 4.6. qRT-PCR Analysis

Total RNA was used for cDNA synthesis using a PrimeScript RT Reagent Kit with a gDNA Eraser (Takara, Kusatsu, Japan). qRT-PCR was performed using a CFX96 Touch Real-Time PCR Detection System (Bio-Rad, Hercules, CA, USA) with a TB Green Premix Ex Taq (Tli RNase H Plus; Takara). Nine differentially expressed genes (Zjn_sc00066.1. g01480.1.sm.mkhc, Zjn_sc00094.1.g00080.1.am.mk, Zjn_sc00002.1.g13730.1.am.mk, Zjn_sc00011.1.g02490.1.sm.mk, Zjn_sc00106.1.g00260.1.am.mk, Zjn_sc00003.1.g09500.1.sm.mkhc, Zjn_sc00008.1.g11130.1.sm.mk, Zjn_sc00058.1.g01990.1.am.mk, and Zjn_sc00023.1.g06490.1.sm.mkhc) were selected for the qRT-PCR analysis. 

The qRT-PCR primers used are listed in Supplementary Table S1. Relative gene expression was calculated using the 2^−ΔΔCt^ method [67]. Each qRT-PCR analysis was performed in triplicate.

## Figures and Tables

**Table 1:** Summary of RNA-seq results in *Z. japonica* under Cd stress treatment.

Sample	Reads No.	Clean Reads No.	Total Mapped	Bases (bp)	Q20 (%)	Q30 (%)
L_0h_1	46,067,222	45,438,370	43,480,195 (95.69%)	6,956,150,522	98.00	94.14
L_0h_2	41,800,042	41,147,778	39,405,918 (95.77%)	6,311,806,342	97.74	93.55
L_0h_3	43,795,346	43,206,630	41,483,551 (96.01%)	6,613,097,246	97.92	93.89
L_6h_1	45,520,700	44,950,190	42,576,734 (94.72%)	6,873,625,700	98.01	94.14
L_6h_2	44,767,754	44,113,820	42,032,965 (95.28%)	6,759,930,854	97.90	93.95
L_6h_3	40,830,954	40,260,512	38,151,253 (94.76%)	6,165,474,054	97.97	94.08
L_12h_1	39,850,416	39,253,016	37,335,934 (95.12%)	6,017,412,816	97.80	93.64
L_12h_2	44,664,070	43,988,038	41,964,721 (95.40%)	6,744,274,570	97.78	93.59
L_12h_3	45,168,508	44,507,012	42,304,429 (95.05%)	6,820,444,708	97.93	94.02
L_24h_1	40,804,266	40,169,930	38,163,038 (95.00%)	6,161,444,166	97.81	93.71
L_24h_2	44,778,440	44,073,644	41,890,835 (95.05%)	6,761,544,440	97.90	93.99
L_24h_3	44,738,228	44,142,912	42,173,022 (95.54%)	6,755,472,428	98.02	94.19

## Data Availability

Data are contained within the article and Supplementary Materials.

## Supplementary Materials

The following supporting information can be downloaded at https://www.mdpi.com/article/10.3390/plants12223833/s1. Figure S1: The correlation between different samples in RNA-seq results; Figure S2: The GO analysis of differentially expressed genes at different time points; Figure S3: The KEGG analysis of differentially expressed genes at different time points; Table S1 Primer sequences for qRT-PCR analysis; Table S2 All the identified common differentially expressed genes; Table S3 GO analysis of common differentially expressed genes; Table S4 KEGG enrichment analysis of common differentially expressed genes; Table S5 Glutathione metabolism related common differentially expressed genes; Table S6 Plant hormone signal transduction related common differentially expressed genes; Table S7 Protein processing in the endoplasmic reticulum pathway related common differentially expressed genes; Table S8 Transport function related common differentially expressed genes; Table S9 Transcription factor related common differentially expressed genes; Table S10 Carbohydrate metabolism related common differentially expressed genes; Table S11 Relative gene expression of selected differentially expressed genes
