# Supplementary figures and images for: Transcriptome Analysis Reveals the Stress Tolerance Mechanisms of Cadmium in Zoysia japonica
Source: Plants (Basel). 2023 Nov 12;12(22):3833. doi: 10.3390/plants12223833 (PMC10674853; doi:10.3390/plants12223833)

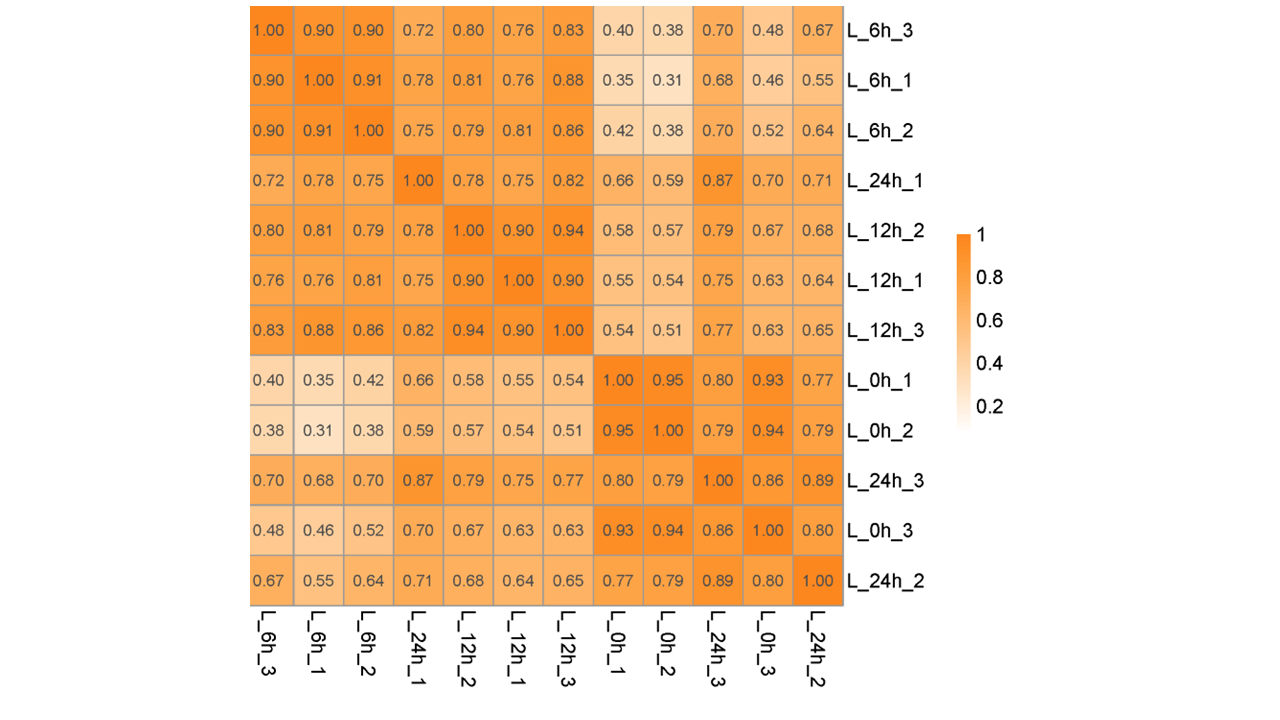

Supplement: Supplementary file 1 [file plants-12-03833-s001.zip › Figure S1.tif]

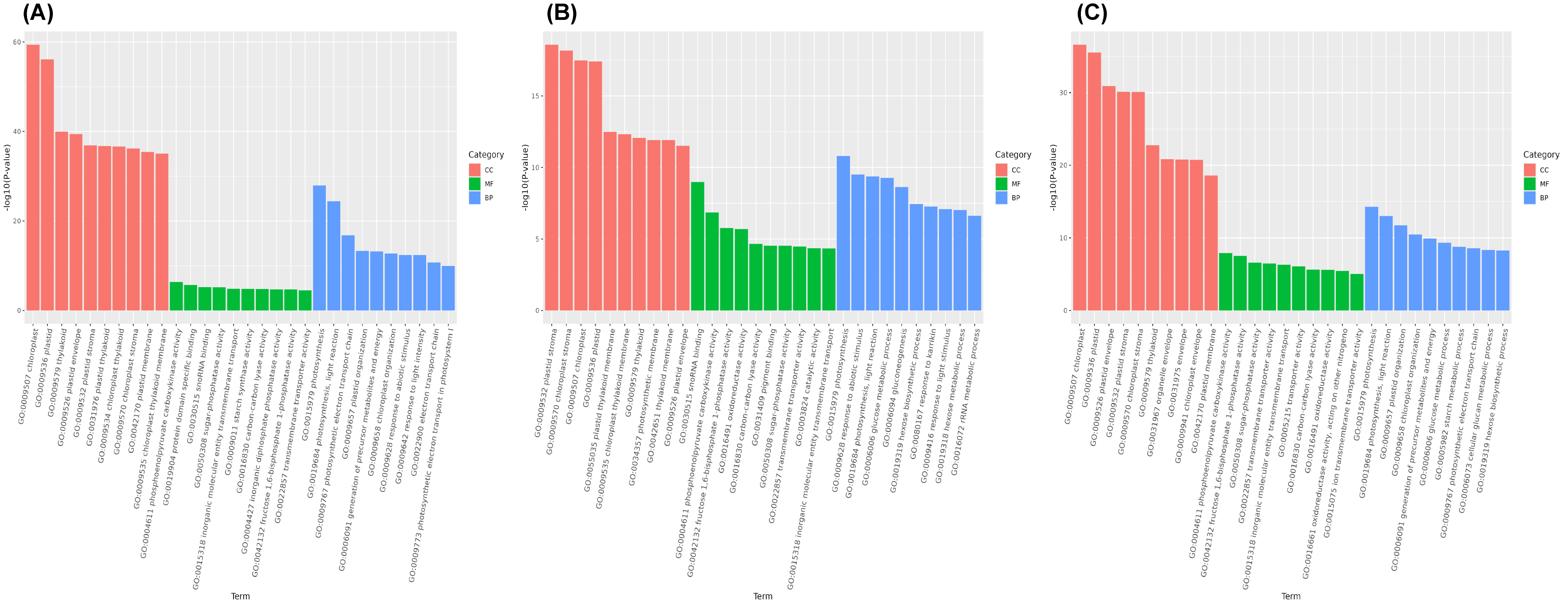

Supplement: Supplementary file 1 [file plants-12-03833-s001.zip › Figure S2.tif]

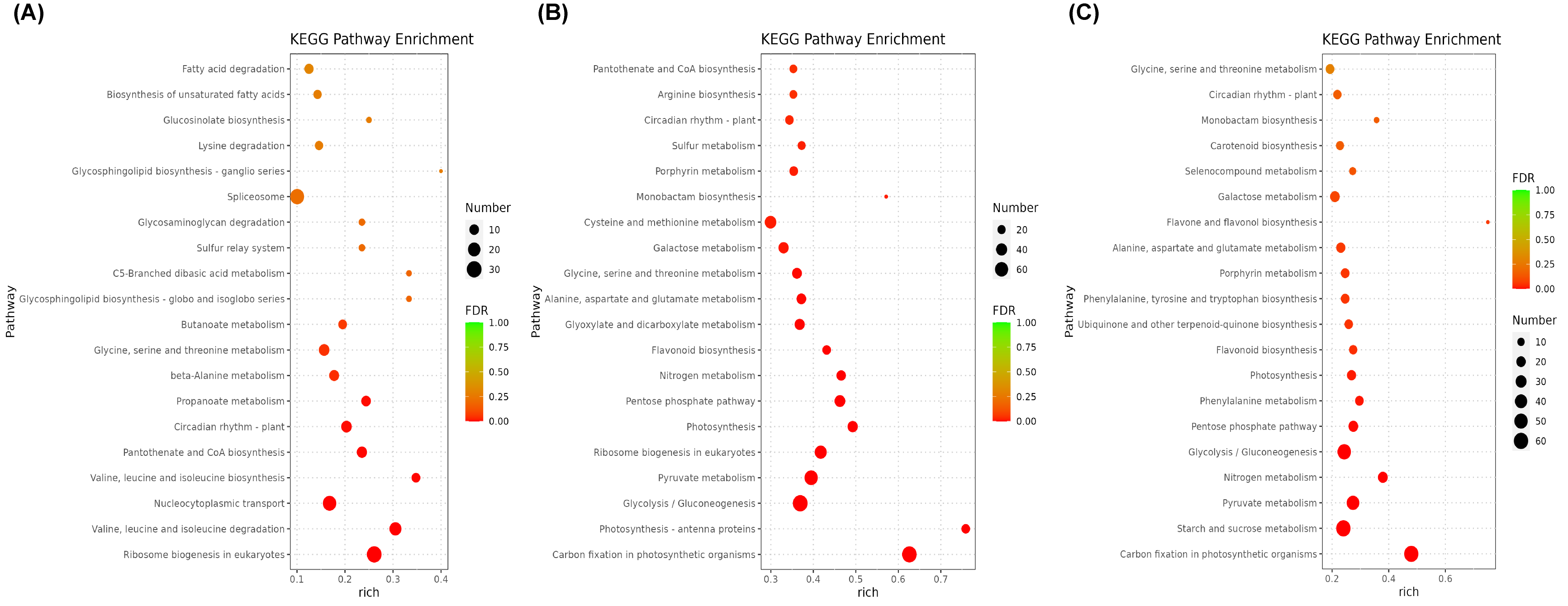

Supplement: Supplementary file 1 [file plants-12-03833-s001.zip › Figure S3.tif]
